# The genome sequence of *Bipolaris cookei* reveals mechanisms of pathogenesis underlying target leaf spot of sorghum

**DOI:** 10.1038/s41598-017-17476-x

**Published:** 2017-12-08

**Authors:** Alex Z. Zaccaron, Burton H. Bluhm

**Affiliations:** Department of Plant Pathology, University of Arkansas, Division of Agriculture, Fayetteville, AR 72701 USA

## Abstract

*Bipolaris cookei* (=*Bipolaris sorghicola*) causes target leaf spot, one of the most prevalent foliar diseases of sorghum. Little is known about the molecular basis of pathogenesis in *B. cookei*, in large part due to a paucity of resources for molecular genetics, such as a reference genome. Here, a draft genome sequence of *B. cookei* was obtained and analyzed. A hybrid assembly strategy utilizing Illumina and Pacific Biosciences sequencing technologies produced a draft nuclear genome of 36.1 Mb, organized into 321 scaffolds with L50 of 31 and N50 of 378 kb, from which 11,189 genes were predicted. Additionally, a finished mitochondrial genome sequence of 135,790 bp was obtained, which contained 75 predicted genes. Comparative genomics revealed that *B. cookei* possessed substantially fewer carbohydrate-active enzymes and secreted proteins than closely related *Bipolaris* species. Novel genes involved in secondary metabolism, including genes implicated in ophiobolin biosynthesis, were identified. Among 37 *B. cookei* genes induced during sorghum infection, one encodes a putative effector with a limited taxonomic distribution among plant pathogenic fungi. The draft genome sequence of *B. cookei* provided novel insights into target leaf spot of sorghum and is an important resource for future investigation.

## Introduction

Sorghum (*Sorghum bicolor* (L.)) was domesticated in northeast Africa 4–5000 years ago, although anthropological records indicate it has been consumed by humans as early as 8000 BC^1,2^. In 2016, the U.S. was the world’s leading producer of sorghum, followed by Nigeria, Sudan, Mexico, India, and China (https://www.worldsorghumproduction.com/previous-year.asp). In the U.S., sorghum is mostly grown as a grain crop, although it is also produced for silage and syrup^3,4^, and has recently garnered interest as a feedstock for cellulosic ethanol production^5^ and in culinary applications due to its gluten-free properties^6^. Sorghum is naturally adapted to harsh growing environments, particularly locations where heat, drought, and marginal soils limit the profitability of crops such as corn or wheat^7^.

**Figure 1:**
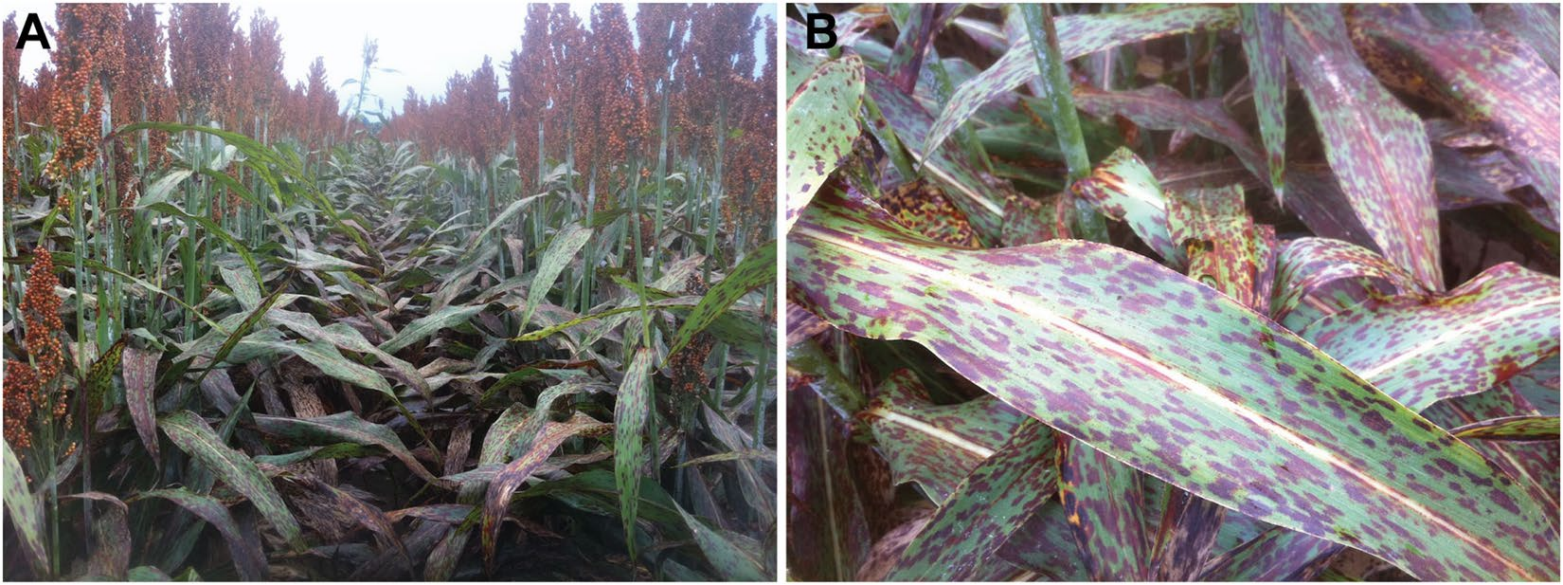
Target leaf spot of sorghum. (**A**) Sorghum plants with symptoms of target leaf spot in the field. (**B**) Necrotic lesions caused by target leaf spot on a sorghum leaf.

Sorghum production, particularly in warm, humid growing areas, is hindered by a wide variety of fungal foliar diseases^8^. Noteworthy in the southeastern U.S. is target leaf spot, caused by *Bipolaris cookei* [=*B. sorghicola* = *Drechslera sorghicola* = *Helminthosporium sorghicola* ^9^]. For many decades after its initial description^10^, target leaf spot was considered to be a disease of minor concern in the U.S.^11^ and other regions of the world^12,13^. However, within two decades after its first reported observation on grain sorghum in Mississippi in 1986^14^, target leaf spot has become widespread in the lower Mississippi river valley. Foliar lesions are distinctly rectangular (Fig. 1A and B), and leaf spots often contain scalariform bands of pigmentation that inspired the disease’s name. The pathogen is generally restrained by the major veins of leaves, but lesions can coalesce under heavy disease pressure, resulting in irregular patches of chlorosis and/or premature leaf death^10^. Most species of *Bipolaris* with defined pathogenicity lifestyles are categorized as necrotrophic pathogens^15^, although hemibiotrophic species have been noted^16^. In *B. cookei*, the growth habit underlying pathogenesis on sorghum has not yet been determined. However, the rapid onset of necrosis after infection, unimpeded intracellular growth during host colonization, and an apparent lack of specialized infectious structures associated with biotrophy (such as infectious hyphae or haustoria) collectively suggest a necrotrophic lifestyle^12^.

The molecular basis of target leaf spot is poorly understood. Quantitative trait loci underlying resistance to target leaf spot have been explored to some extent^17,18^. A single recessive resistance gene (*ds1*) was positionally cloned and postulated to encode a leucine-rich receptor kinase family protein^19,20^. Comparative transcriptomics during infection of resistant and susceptible hybrids with *B. cookei* identified both plant and fungal genes 12 and 24 hr after infection^21,22^. However, only 160 transcripts were attributed to *B. cookei*, likely due to the low amount of fungal biomass and difficulty mapping transcripts due to the lack of a reference genome sequence for the pathogen.

To address the lack of genomic resources of *B. cookei* and provide new insights into the mechanistic basis of target leaf spot, a draft genome assembly of *B. cookei* was assembled and analyzed. Broad categories of genes involved in pathogenesis, such as carbohydrate-active enzymes and genes involved in secondary metabolism, were identified and assessed through comparative genomics. Additionally, novel genes were identified that could play important roles in pathogenicity.

## Results

### Genome sequencing, assembly, and annotation

The *B. cookei* genome was sequenced with Illumina and Pacific Biosciences (PacBio) sequencing technologies. Illumina sequencing produced 176,463,008 reads (2 × 100 bp paired-end sequences), and PacBio sequencing produced 251,565 reads with lengths of 35 to 21,246 bp (average length = 2,427 bp). A hybrid assembly pipeline (Supplementary Fig. S1) incorporated both types of reads to produce a draft genome assembly of 36,171,030 bp organized into 321 scaffolds (average length = 112 kb) (Table 1).

**Table 1:** Genome assembly statistics of *B. cookei* compared with other *Bipolaris* species.

	*B. cookei*	*B. maydis* C5	*B. sorokiniana*	*B. zeicola*	*B. oryzae*	*B. victoriae*
Genome size (bp)	36,171,030	36,456,735	34,417,436	31,267,936	31,362,097	32,829,575
Scaffolds	321	68	157	844	619	676
Contigs	533	88	507	882	671	714
Average scaffold length (bp)	112,682	536,128	219,219	37,047	50,665	48,564
Scaffold L50	31	7	7	82	68	47
Scaffold N50 (bp)	378,688	1,842,487	1,789,485	110,153	134,117	231,353
Longest scaffold (bp)	1,437,825	4,213,224	3,642,493	501,469	638,489	870,365
GC content (%)	49.9	49.7	49.8	50.8	50.5	50.1
Gap (%)	0.5	0.3	3.5	0.0	0.1	0.0

Approximately 14% of the *B. cookei* draft genome sequence was comprised of transposable elements (TEs). Of these, DNA transposons (class 2 TEs) corresponded to 9% of the *B. cookei* genome, and retrotransposons (class 1 TEs), comprised 3% (Supplementary Fig. S2). The majority of the classified DNA transposons contained a DDE superfamily transposase motif^23^, which comprised approximately 3 Mb of the draft assembly.

To assist gene prediction, RNA-seq was performed from *in vitro* cultures of *B. cookei*. RNA was sequenced with Ion Torrent technology, which generated 6,273,768 reads (average length = 247 bp). After RNA-seq reads were mapped to the *B. cookei* genome assembly, 17,998 transcripts were reconstructed, corresponding to 11,451 distinct loci. With reconstructed transcripts and proteins from closely related species as evidence, 11,189 distinct protein-encoding genes were predicted (average length = 1707 bp) (Table 2). Assessment of predicted genes with BUSCO software showed 95.7% gene completeness, with 0.2% duplication, and 2.9% fragmentation. Functional characterization of the predicted *B. cookei* proteins revealed that 10,801 proteins (96%) had at least one homologous sequence in the NCBI nr database (e-value < 1e-5), 8,422 proteins (75%) had a conserved domain matching the InterPro database, and 9,034 proteins (80%) had a GO term attributed by Blast2GO.

**Table 2:** Gene prediction statistics of *B. cookei* and other *Bipolaris* species.

	*B. cookei*	*B. maydis C5*	*B. sorokiniana*	*B. zeicola*	*B. oryzae*	*B. victoriae*
Genes	11,189	13,336	12,250	12,857	12,007	12,894
Average length of:						
Genes (bp)	1,707	1,580	1,691	1,418	1,479	1,432
ORFs (bp)	1,580	1,439	1,474	1,418	1,479	1,432
Proteins (aa)	465	427	443	427	445	431
Introns (bp)	99	97	91	87	88	88
Exons (bp)	493	535	587	505	416	411
Gene completeness	95.7%	97.6%	98.2%	97.0%	97.1%	96.9%
Duplicated genes	0.2%	2.4%	0.2%	0.2%	0.2%	0.2%
Fragmented genes	2.9%	1.5%	0.8%	1.0%	0.9%	0.8%
Missing genes	1.4%	0.9%	1.0%	2.0%	2.0%	2.3%

Homology-based analyses with mating-type loci from related *Bipolaris* species indicated that *B. cookei* strain LSLP18.3 contained a *MAT-1* gene (Bc_02106). The predicted protein encoded by Bc_02106 shared 87% amino acid identity with *MAT-1* from *B. maydis*, and was located within a genomic region conserved between *B. maydis* and *B. cookei* (Supplementary Fig. S3).

**Figure 2:**
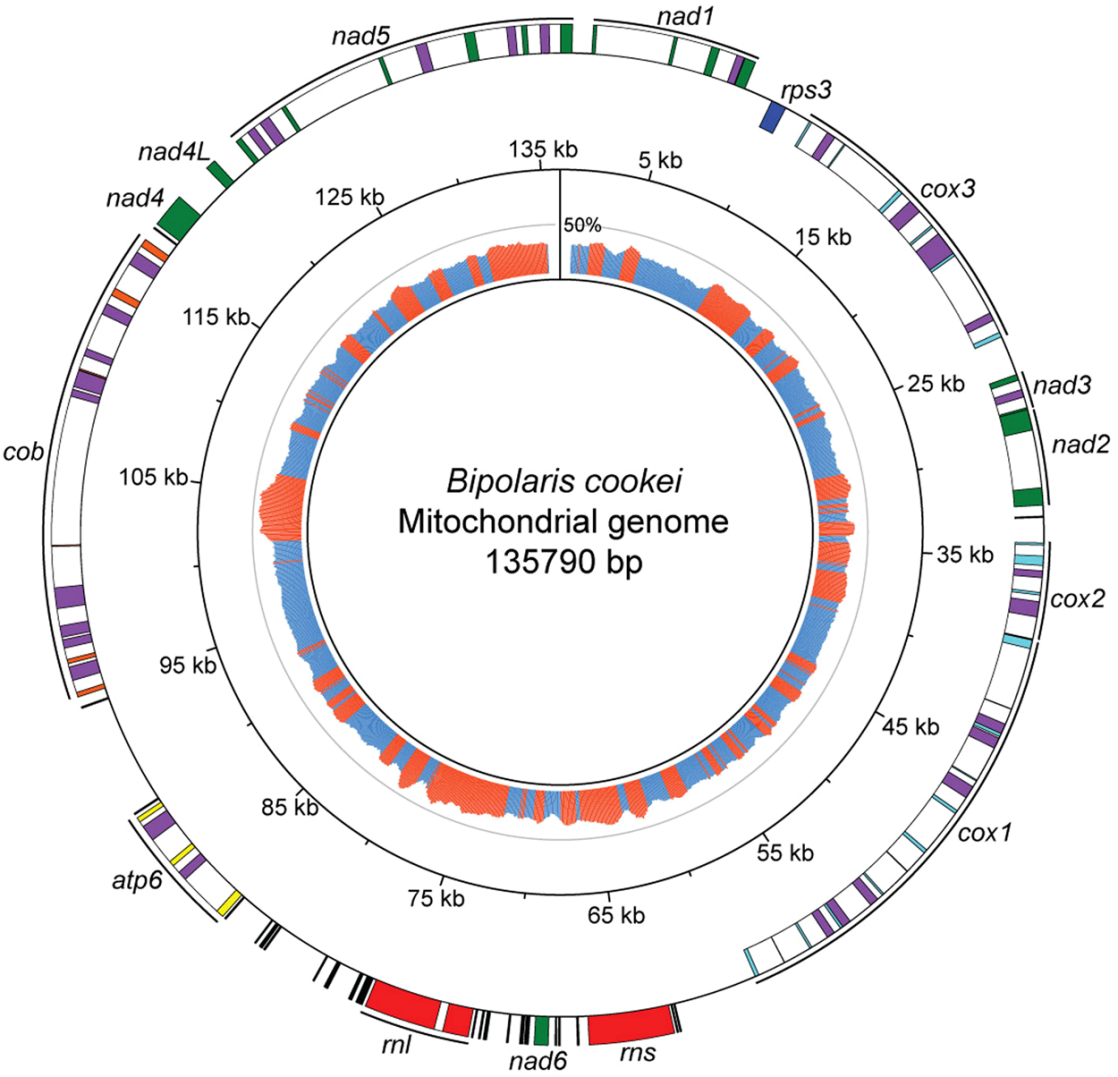
Circular representation of the *B. cookei* mitochondrial genome. Mitochondrial genes are represented as rectangles on the outermost circle. Genes on the forward and reverse strand are drawn outward and inward, respectively. Genes encoding subunits of the electron transport chain of complex I (*nad1–6*) are in green, complex III (*cox1–3*) in cyan, complex IV (*cob*) in orange, ATP synthase (*atp*6) in yellow, rDNAs (*rns* and *rnl*) in red, tRNAs in black and ribosomal protein S3 (*rps3*) in blue. ORFs encoding homing endonucleases are drawn in purple, and introns are represented as white regions. The innermost circle represents the GC content, calculated with a sliding window of 1 kb and step size of 100 bp. Regions with the GC content below and above the average (30%), are in blue and orange, respectively. A grey circle designates the 50% GC content.

In addition to the nuclear genome, the *B. cookei* mitochondrial genome was assembled as a circular sequence of 135,790 bp with a GC content of 30% (Fig. 2). A total of 75 mitochondrial genes were predicted, including 12 of 14 highly conserved genes among fungal mitochondria: four subunits of the respiratory chain complexes (*cox1*, *cox2*, *cox3* and *cob*), seven NADH dehydrogenase subunits (*nad1*, *nad2*, *nad3*, *nad4*, *nad4L*, *nad5* and *nad6*), and one ATP synthase (*atp6*)^24^. Additionally, 30 genes encoding tRNAs were predicted, which were able to recognize all 20 standard amino acids; 2 rDNAs (small and large subunits); a gene encoding ribosomal protein S3 (*rps3*); and 30 ORFs predicted to encode homing endonucleases (21 from the LAGLIDADG family and 9 from the GIY-YIG family) located within intronic regions. The *B. cookei* mitochondrial genome assembly contained 36 introns (average length = 2,284 bp), which comprised a total of 82,220 bp. Comparative genomic analyses with the mitochondrial gene *cob* from *Cercospora beticola* ^25^ indicated that the mutation G143A, which is associated with resistance to QoI fungicides, is not present in *B. cookei* strain LSLP18.3 (Supplementary Fig. S4).

### Carbohydrate-active enzymes (CAZymes)

A total of 589 genes encoding CAZymes were identified in the *B. cookei* genome (Table 3; Supplementary Table S1). The three most populated CAZyme families were CE10 with 38 genes, AA7 with 32 genes, and CE1 with 26 genes. Both CE10 and CE1 families include a great variety of esterases, such as acetyl xylan esterases (EC: 3.1.1.72), carboxylesterases (EC: 3.1.1.1), and acetylcholinesterases (EC: 3.1.1.7)^26^, whereas family AA7 includes gluco- and chitooligosaccharide oxidases (EC: 1.1.3.-)^27^.

**Table 3:** Number of genes encoding carbohydrate-active enzymes across different *Bipolaris* species.

	GH	GT	PL	CE	CBM	AA	Total
*B. cookei*	238	95	16	105	52	115	589
*B. maydis* C5	279	102	15	135	66	145	705
*B. zeicola*	264	95	15	129	63	130	656
*B. oryzae*	259	98	15	124	58	131	648
*B. sorokiniana*	260	99	15	125	66	125	651
*B. victoriae*	267	95	15	131	67	130	664

GH: glycoside hydrolases; GT: glycosyltransferases; PL: polysaccharide lyases; CE: carbohydrate esterases; CBM: carbohydrate-biding modules; and AA: auxiliary activities.

**Figure 3:**
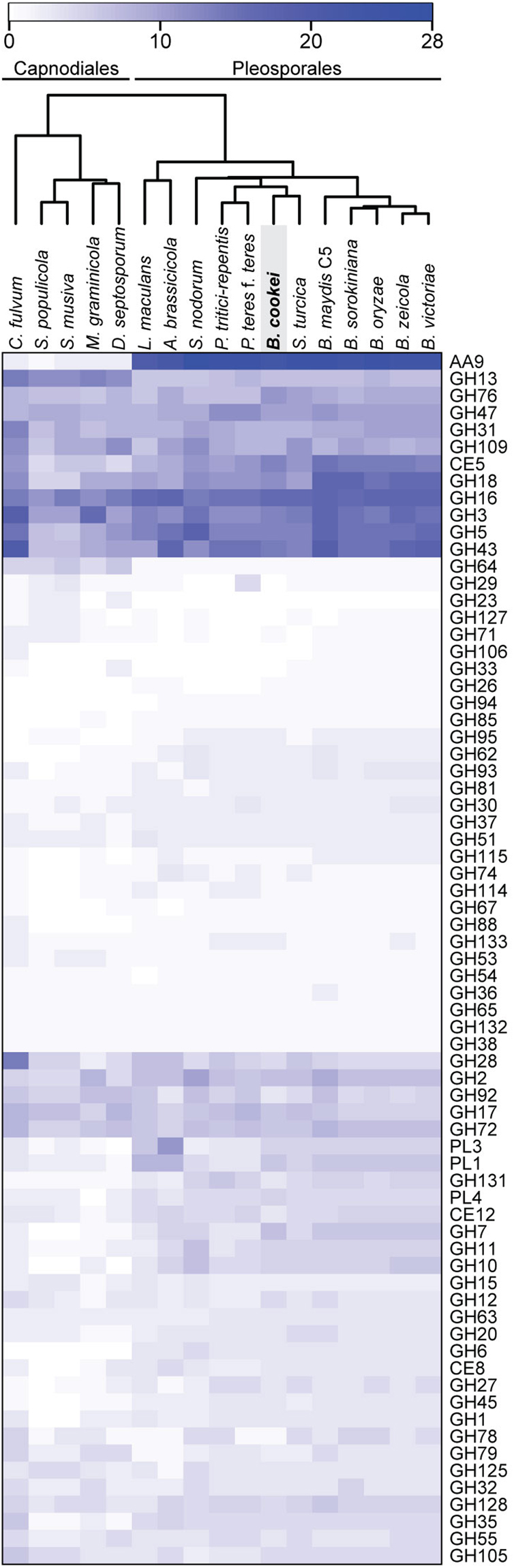
Heatmap showing the number of genes encoding glycoside hydrolases (GHs) and other CAZyme families that include plant cell wall degrading enzymes. *B. cookei* and 16 other members of the Dothideomycetes were hierarchically clustered. The full name of each species is provided in Supplementary Table S1.

CAZymes categorized as glycoside hydrolases (GHs) receive particular attention for their ability to hydrolyse glycosidic bonds between carbohydrates or between a carbohydrate and a non-carbohydrate moiety^26^. In *B. cookei*, the most populated GH families were GH16 with 16 genes, GH43 with 14 genes, and GH3 with 13 genes (Fig. 3). Family GH16 is comprised of numerous endoglucanases, which catalyze hemicellulose degradation^28^. Family GH43 includes enzymes such as xylanases, α-L-arabinofuranosidases, and β-D-galactosidases, that debranch and degrade hemicellulose and pectin^29^. Most *B. cookei* CAZymes from family GH3 were functionally annotated as β-glucosidases (EC: 3.2.1.21). Beta-glucosidases play an important role in the degradation of cellulose by converting cellobiose into glucose^30^.

Many members of the AA class of CAZymes are carbohydrate oxidases that assist other enzymes from GH, PL and CE classes to gain access to carbohydrates present in plant cell walls^31^. Two-thirds of the AA CAZymes in the *B. cookei* genome belonged to families AA7 (32 genes), AA9 (23 genes), and AA11 (11 genes). While family AA7 includes gluco- and chitooligosaccharide oxidases, family AA9 is represented by lytic polysaccharide monooxygenases (LPMOs) that help cellulases and hemicellulases break down cellulose and hemicellulose^32^. CAZymes from family AA9 often act in conjunction with cellobiose dehydrogenases (CDHs), found in family AA3, to accelerate oxidative degradation of cellulosic materials, including foliar tissues of plants^32–34^.

*B. cookei* and 16 other plant pathogenic members of the Dothideomycetes were clustered based on the number of CAZymes belonging to families likely involved in plant cell wall degradation. In concordance with previous studies, species within the Capnodiales and Pleosporales were organized in two distinct branches that resemble their phylogenetic placement^35–37^ (Fig. 3). The number of enzymes from families AA9 (LPMOs), GH64 (β-1,3-glucanases), CE5 (cutinases), PL1, and PL3 (pectin lyases) were notably different between Capnodiales and Pleosporales members. The number of GH13 enzymes, which include α-amylases and related enzymes involved in starch degradation^38^, was also markedly different. On average, members of the Capnodiales had 12.6 proteins from family GH13, whereas members of the Pleosporales had 6.9. Interestingly, hierarchical clustering grouped all analyzed *Bipolaris* spp. together in a distinct branch with the exclusion of *B. cookei*. *B. cookei* instead grouped most closely with *Setosphaeria turcica* in a different branch that also included *Pyrenophora tritici-repentis*, *P. teres* f. *teres*, and *Stagonospora nodorum* (Fig. 3).

### Secondary metabolism genes

**Figure 4:**
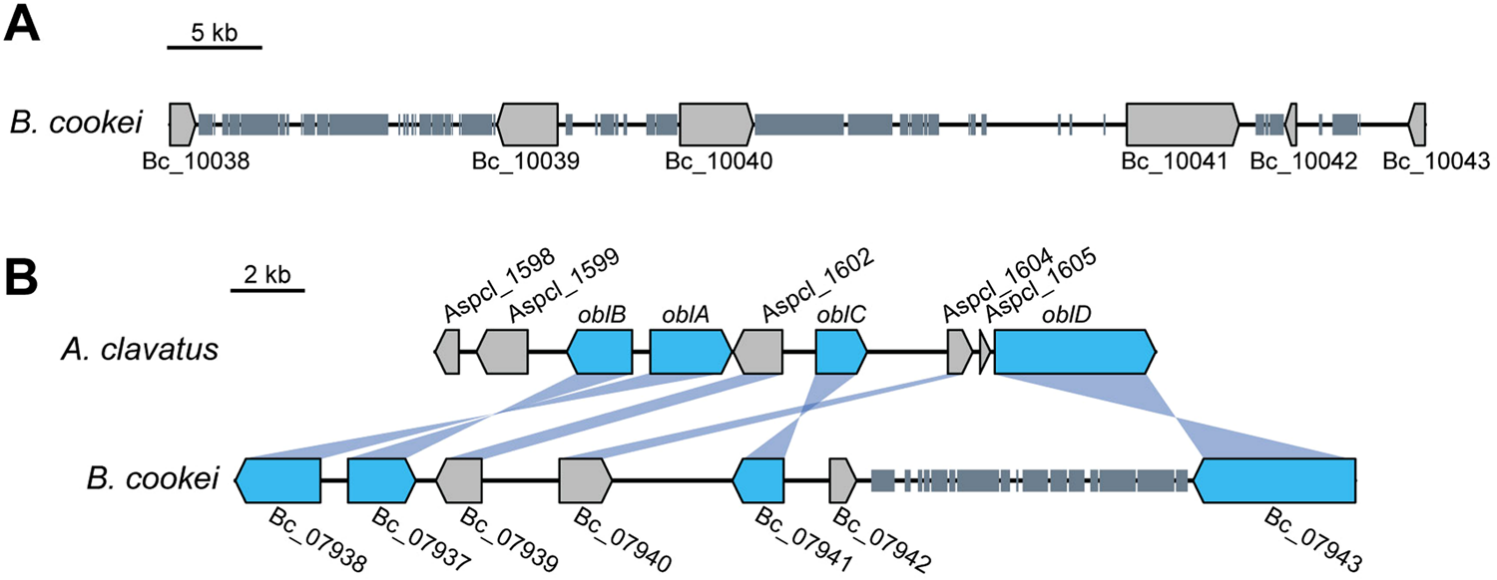
Secondary metabolism genes. (**A**) Genomic region around *B. cookei* gene Bc_10041, a PKS-encoding gene possibly acquired from *Colletotrichum sublineolum* via horizontal transfer. Gene Bc_10038 encodes a putative alpha-xylosidase, Bc_10039 and Bc_10040 both encode NRPSs, Bc_10042 and Bc_10043 are hypothetical genes. (**B**) Ophiobolin gene cluster in *Aspergillus clavatus* and the corresponding homologous cluster in *B. cookei*. *A. clavatus* genes that are part of the cluster are labelled *oblA*-*oblD*, and other genes are labelled with the JGI protein ID. *oblA* encodes a terpene synthase, *oblB* encodes a cytochrome P450, *oblC* encodes a flavin-dependent oxidase, and *oblD* encodes a major facilitator transport protein. Genes Bc_07939, Bc_07940, and Bc_07942 encode hypothetical proteins. Bc_07939 has a bZIP domain, and Bc_07942 has a NAD(P)-binding domain. Repetitive DNA is represented as small rectangles.

Plant pathogenic fungi possess a diverse array of genes involved in the biosynthesis of secondary metabolites (SMs). SMs are often produced by genes organized into clusters that contain one or more genes referred to as backbone genes, which are primarily classified as polyketide synthases (PKSs), non-ribosomal peptide synthetases (NRPSs), hybrid PKS-NRPSs, dimethylallyl tryptophan synthases (DMATs), or terpene synthases (TSs)^39^. *B. cookei* had 47 backbone genes involved in secondary metabolism, categorized as 20 PKSs, 17 NRPSs, 3 DMATs, and 7 TSs, which is consistent with other *Bipolaris* species analyzed so far (Supplementary Table S2). One of the PKSs was a type III PKS, and the remaining 19 were type I PKSs, which were further classified into 10 highly reducing (HR-PKS), 2 partially reducing (NR-PKS), and 7 non-reducing (NR-PKS) (Supplementary Table S3). Orthologs of one NR-PKS from *B. cookei* (Bc_10041) could not be identified in the genomes of other *Bipolaris* species via homology searches (Supplementary Table S4), and the most similar protein identified among all fungi corresponded to a hypothetical gene from another sorghum pathogen, *Colletotrichum sublineolum* (55% amino acid identity). Gene Bc_10041 was located in a genomic region enriched with repetitive DNA, in close proximity to two other backbone genes encoding NRPSs, Bc_10039 and Bc_10040 (Fig. 4A). Interestingly, putative orthologs of Bc_10039 were not detected in the genomes of other *Bipolaris* species, and Bc_10040 was orthologous to *NPS9* (78% identity), a NRPS-encoding gene from *B. maydis* that was previously thought to be unique to this species^15^ (Supplementary Table S4).

Phytotoxic sesquiterpenoids of the ophiobolin family have reportedly been produced by *B*. *cookei* and other *Bipolaris* species^40–42^. Recently, the gene cluster responsible for ophiobolin F biosynthesis was characterized in *Aspergillus clavatus* ^43^. Based on this report, a similar gene cluster was identified in *B*. *cookei* that contained homologs of the terpene synthase *oblA* (Bc_07938, 62% identity), the cytochrome P450 *oblB* (Bc_07937, 68% identity), the FAD dependent oxidoreductase *oblC* (Bc_07941, 52% identity), and the ABC transporter *oblD* (Bc_07943, 76% identity) (Fig. 4B). Two additional ORFs present in the *A. clavatus* ophiobolin cluster were also conserved in *B. cookei*: a homolog of Aspc_1602 (Bc_07939, 38% identity; a hypothetical protein with no conserved domains), and a homolog of Aspcl_1604 (Bc_07940, 43% identity; a putative bZIP transcription factor). The additional ORF in the *B. cookei* ophiobolin cluster that is not present in the *A. clavatus* cluster (Bc_07942) encoded a hypothetical protein containing a NAD(P)-binding domain.

The PKS-encoding gene Bc_07091 of *B. cookei* was highly homologous to *PKS18* of *B. maydis* (98% amino acid identity), which is involved in melanin biosynthesis^44^. The draft genome sequence of *B. cookei* contained additional genes involved in melanin biosynthesis, including the 1,3,8-trihydroxynaphthalene reductase *BRN1* (Bc_07086, 92% identity), the transcription factor *CMR1* (Bc_07088, 97% identity), the 1,3,6,8-tetrahydroxynaphthalene reductase *BRN2* (Bc_07555, 99% identity), and the scytalone dehydratase *SCD1* (Bc_10431, 98% identity) (Supplementary Fig. S5).

### Secretome and candidate effectors

A total of 1035 genes were identified in the *B. cookei* nuclear genome that encoded proteins containing a signal peptide for secretion. Among these genes, 170 were predicted to encode proteins containing transmembrane domains or a glycosylphosphatidylinositol (GPI) anchor (Supplementary Table S5), and thus were predicted to be cell surface proteins^45^. The remaining 865 proteins were determined to comprise the *B. cookei* secretome. Nearly half (42%) of the predicted secretome consisted of proteins implicated in aspects of primary metabolism. Specifically, the putative secretome contained 253 CAZymes, with AA families being the most abundant: 22 proteins from family AA9, 18 proteins from family AA7, and 15 proteins from family AA3. Additionally, 74 proteases, 43 peroxidases, and 107 lipases were identified in the *B. cookei* secretome (Supplementary Table S5).

A set of 233 proteins were classified as candidate effectors, or small secreted proteins (SSPs). Among these, 133 could not be assigned a putative function based on homology, and 149 had no conserved domains. One of the *B. cookei* SSPs (Bc_04981) was homologous to *Ecp6* from the tomato pathogen *Cladosporium fulvum* ^46^. *Ecp6* is an effector that contains lysin motif (LysM) domains, and sequesters chitin oligosaccharides released from the fungal cell wall to avoid host chitin-triggered immune responses^46^. The architecture of the three LysM domains found in *Ecp6* was conserved in the *B. cookei* homolog Bc_04981 (Supplementary Fig. S6)

### Expression-based analyses to identify candidate genes involved in pathogenesis

Fungal genes preferentially expressed during host infection are potentially involved in pathogenicity. To qualitatively identify *B. cookei* genes highly or exclusively expressed *in planta*, an RNA-seq data set was obtained from *B. cookei* grown on various defined culture media *in vitro*, representing the basal transcriptome, which was then compared to *in planta* expression data from sorghum leaves infected by *B. cookei* ^21^ (accessions: DRR006371 and DRR006373). Of the 66 million reads obtained by Yazawa and co-authors^21^, 537,582 (0.8%) mapped to the *B. cookei* draft genome assembly. Fungal RNA-seq reads identified *in planta* accounted for 54,295,782 bp, as compared to 1,422,960,112 bp (5,531,920 reads) obtained *in vitro*. A total of 37 genes were considered to be induced *in planta*, 16 of which had no evidence of expression *in vitro* (Table 4). Several of the identified genes induced *in planta* (e.g. oxidoreductase activity enzymes, major facilitator superfamily transporters, and SM backbone genes) were associated with fungal secondary metabolite biosynthesis or detoxification of compounds. Additionally, four genes encoding candidate effectors were also considered to be induced *in planta*.

**Table 4:** *B. cookei* genes considered induced during infection.

Gene ID	ORF size (bp)	Reads *in panta*	Reads *in vitro*	Secreted	Blast2GO functional description
Bc_10331	456	421	0	NO	Snoal-like polyketide cyclase family 2
Bc_11188	1455	259	0	NO	6-hydroxy-d-nicotine oxidase
Bc_10330	1192	226	0	NO	Dehydrogenase reductase SDR family member 7B
Bc_10329	1618	176	0	NO	Cytochrome P450
Bc_04819	1894	78	0	NO	X-Pro dipeptidyl-peptidase (S15 family)
Bc_06468	415	70	0	YES	Hypothetical protein
Bc_06321	1724	43	0	NO	Glucooligosaccharide oxidase
Bc_04208	915	12	0	NO	Hypothetical protein
Bc_04302	309	11	0	NO	Hypothetical protein
Bc_09129	537	8	0	NO	dj-1 family
Bc_09118	483	8	0	NO	Mitochondrial carrier
Bc_09119	1290	8	0	NO	Mitochondrial carrier
Bc_04701	512	7	0	YES	Hypothetical protein
Bc_03870	1924	6	0	YES	Glutamyl-tRNA(Gln) amidotransferase subunit A
Bc_00034	1853	5	0	NO	Cobalamin synthesis
Bc_11121	1898	4	0	NO	High affinity methionine permease
Bc_01083	1468	9	2	No	Major facilitator superfamily transporter
Bc_01920	2277	363	36	No	Catalase A
Bc_03407	1789	14	1	No	Dibenzothiophene desulfurization enzyme A
Bc_04579	7882	6	1	No	Polyketide synthase
Bc_04816	1671	67	3	No	Aldehyde dehydrogenase
Bc_04818	1057	79	2	No	Aromatic ring-opening dioxygenase family
Bc_04820	1176	110	9	No	Galactonate dehydratase
Bc_05113	497	147	27	No	Cupin domain containing
Bc_06848	651	207	28	Yes	Hypothetical protein
Bc_06913	2635	24206	4976	No	Heat shock 70 kda
Bc_07074	345	11	2	Yes	Hypothetical protein
Bc_08665	984	12	1	No	Acetate transporter
Bc_09116	231	8	2	No	Hypothetical protein
Bc_09501	1800	14	3	No	Major facilitator superfamily transporter
Bc_09836	3398	92	6	No	Salicylate hydroxylase
Bc_10039	3324	4	1	No	Non-ribosomal peptide synthetase
Bc_10847	1047	37	1	No	Cinnamyl-alcohol dehydrogenase
Bc_10957	930	47	1	Yes	Dimeric alpha-beta barrel
Bc_11123	1532	33	8	No	Pisatin demethylase
Bc_11127	1135	30	1	No	Gentisate 1,2-dioxygenase
Bc_11189	1846	7	1	No	Cytochrome P450

The number of *in planta* and *in vitro* RNA-seq reads mapped to each gene ORF is shown. *In planta* RNA-seq reads were sequenced from sorghum leaves infected by *B. cookei* ^21^, and *in vitro* RNA-seq reads were sequenced from *B. cookei* grown on various culture media. Genes with evidence of expression only *in planta* (at least four reads), or with a ratio of reads *in planta*/reads *in vitro* of at least four, were considered induced during infection.

**Figure 5:**
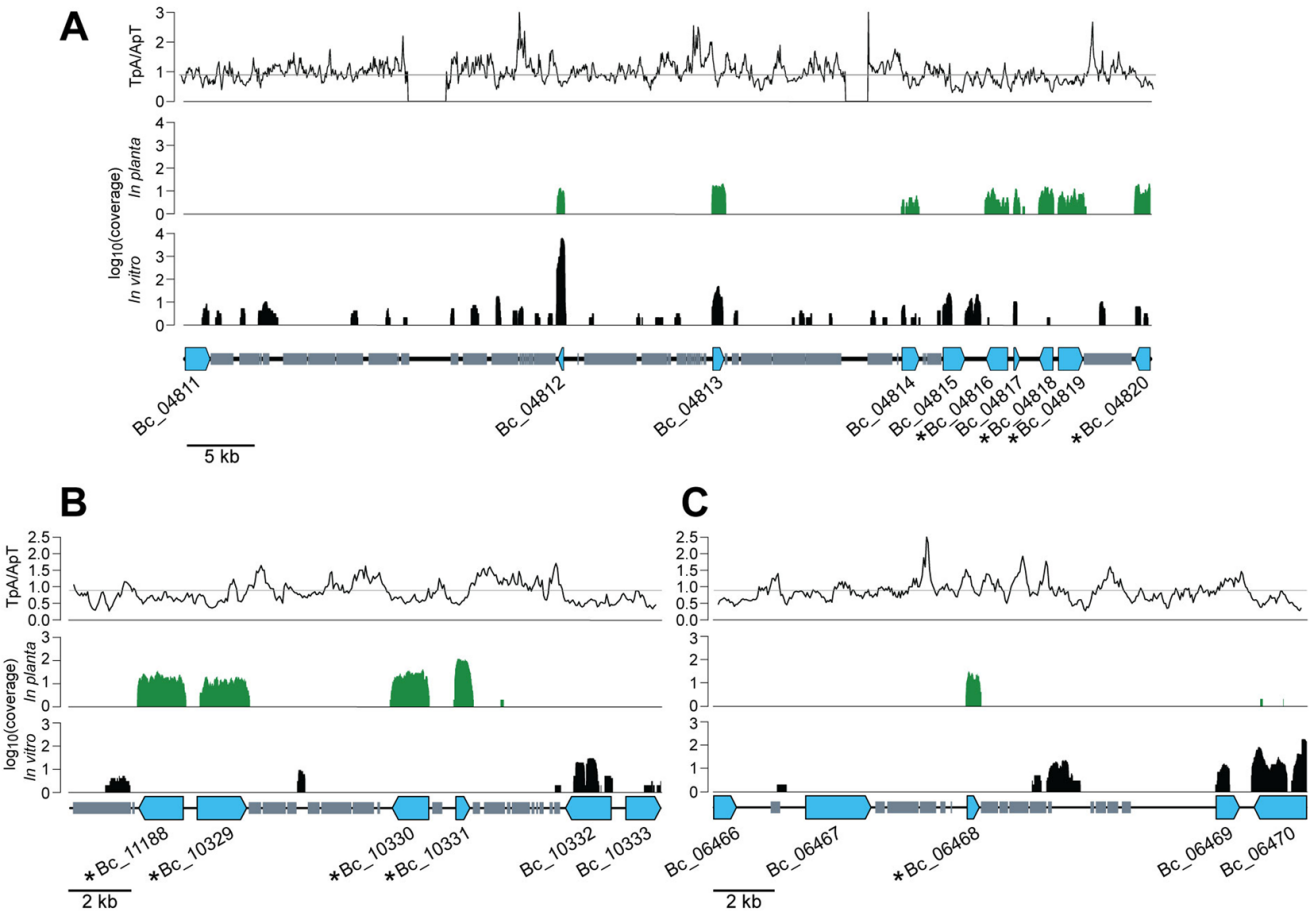
Regions of the *B*. *cookei* genome containing genes induced during sorghum infection. *B. cookei* genes designated as induced during early stages of sorghum infection are indicated with “*”. Putative functions of the genes are (**A**) aldehyde dehydrogenase (Bc_04816), aromatic ring-opening dioxygenase (Bc_04818), X-Pro dipeptidyl-peptidase S15 (Bc_04819), and galactonate dehydratase (Bc_04820). (**B**) 6-hydroxy-d-nicotine oxidase (Bc_11188), cytochrome P450 (Bc_10329), short-chain dehydrogenase reductase (Bc_10330), snoal-like polyketide cyclase (Bc_10331). (**C**) Candidate effector (Bc_06468). Plots above the genes represent the RNA-seq coverage *in vitro* and *in planta* (logarithmic scale), and the RIP index (TpA/ApT) calculated with a sliding window of 300 bp and step size of 50 bp. The minimum RIP index considered as evidence of RIP (0.89)^104^ is marked with a horizontal grey line. Repetitive DNA is represented as small grey rectangles.

Interestingly, some of the genes induced *in planta* were clustered in two regions of the *B. cookei* genome (Fig. 5A and B). Both regions were rich in repetitive DNA, and contained signs of repeat-induced point (RIP) mutations. One region (Fig. 5B) contained four genes exclusively expressed *in planta*. These four genes corresponded to a CAZyme from family AA7, annotated as 6-hydroxy-d-nicotine oxidase (Bc_11188), a putative cytochrome P450 (Bc_10329), a putative dehydrogenase reductase (Bc_10330), and a protein containing a nuclear transport factor 2 superfamily domain, annotated as SnoaL-like polyketide cyclase family 2 (Bc_10331). Homology searches revealed that these four genes were not conserved in other *Bipolaris* species or members of the Pleosporaceae family (Supplementary Table S4). Additionally, the closest homolog of Bc_10331 was a hypothetical protein from the sorghum pathogen *C. sublineolum* (67% amino acid identity).

Additional genes with exclusive *in planta* expression evidence included a serine protease (Bc_04819), an additional AA7 CAZyme (Bc_06321), and two candidate effectors (Bc_06468 and Bc_04701) (Table 4). Bc_06468 was located within a genomic region rich in repetitive DNA that showed signs of RIP mutation (Fig. 5C), and was classified as a putative effector due to its small size (98 amino acids), predicted signal peptide for secretion, and lack of conserved domains. Interestingly, homologs of Bc_06468 were found only in *B. maydis*, *B. zeicola*, *B. victoriae*, *B. sorokiniana*, *B. oryzae*, *S. turcica*, *P. tritici-repentis*, *Fusarium oxysporum* and *F. oxysporum* f. sp. *cubense* (Supplementary Table S4).

The PKS-encoding gene exclusively expressed *in planta* (Bc_04579) was homologous to *alt5* (82% identity), a PKS gene from *Alternaria solani* postulated to be involved in alternapyrone biosynthesis^47^. Homologs of other genes also predicted to be involved in alternapyrone biosynthesis were clustered with Bc_04579, including Bc_11190 (82% identity with the cytochrome P450 *alt2*), Bc_11189 (87% identity with the cytochrome P450 *alt3*), and Bc_04580 (76% identity with the FAD-dependent oxygenase/oxidase *alt4*) (Supplementary Fig. S7). Interestingly, these four genes were located on a 30 kb region of the *B. cookei* genome with little or no evidence of expression *in vitro* (Supplementary Fig. S8). This genomic region also contained four other genes predicted to encode an NAD-dependent epimerase (Bc_04582), a major facilitator superfamily transporter (Bc_04583), an acetylcholinesterase (Bc_04584), and a hypothetical protein (Bc_04585).

### Enrichment of repetitive DNA in proximity to pathogenicity-related genes

**Figure 6:**
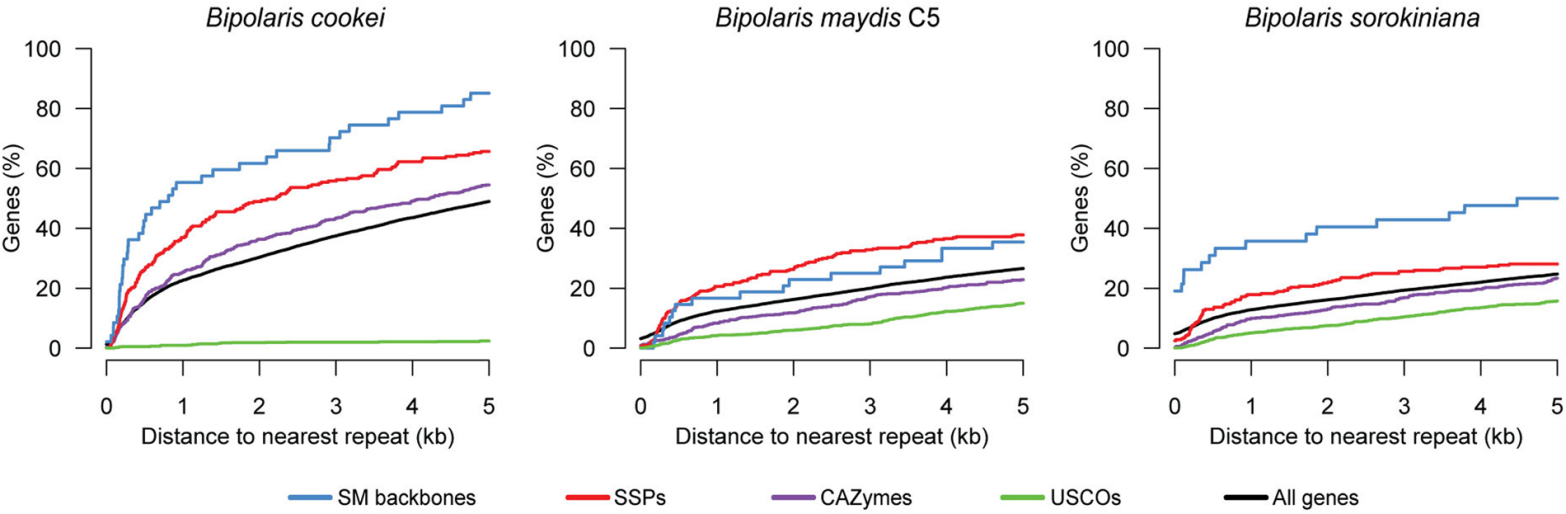
Proximity of classes of genes to repetitive elements. The charts show the fraction of genes from *B. cookei*, *B. maydis* C5, and *B. sorokiniana* that have a repetitive element within an adjacent region up- or downstream of their ORFs of size shown on the horizontal axis. SM backbones: secondary metabolite backbone genes (PKSs, NRPSs, TPs, hybrid PKS-NRPSs, DMATs, and TPs); SSPs: small secreted proteins (candidate effectors); CAZymes: carbohydrate-active enzymes; USCOs: universal single-copy orthologs among members of the Acomycota^88^.

In some plant pathogenic fungi, genes important for pathogenicity are in close proximity to repetitive genomic regions^35^. Genes co-localized with repetitive elements are postulated to provide pathogens an evolutionary advantage due to allelic diversification induced by RIP activity. Consistent with other plant pathogenic fungi^35^, candidate effectors and backbone genes for secondary metabolite production in *B. cookei* were localized in closer proximity to repetitive elements than the overall predicted genes and genes highly conserved among members of the Ascomycota (Fig. 6). More specifically, nearly all *B. cookei* backbone genes (40 out of 47; 85%) and most candidate effectors (153 out of 233; 65%) had repetitive DNA within 5 kb up- or downstream of their ORFs, while about half of all predicted genes (5,478 out of 11,189; 49%) had an analogous arrangement. We also observed similar enrichment of repeats near SM backbone genes and SSPs in the close relatives *B. maydis* C5 and *B. sorokiniana*, albeit not as pronounced as in *B. cookei* (Fig. 6). Interestingly, only 26% and 24% of the *B. maydis* C5 and *B. sorokiniana* predicted genes, respectively, had a repeat within a neighbor region of 5 kb, which is much less than the ratio in *B. cookei* (49%). These results indicate that *B. cookei* has a substantial enrichment of repetitive elements near genes potentially important for pathogenicity, and that repeats are more dispersed in the *B. cookei* genome as compared to closely related species.

## Discussion

The draft genome sequence of *B. cookei* strain LSLP18.3 expanded comparative genomics analyses among members of the order Pleosporales within the Dothidiomycetes class of fungi. To date, the genomes of at least 60 members of the Pleosporales have been sequenced^48^. Although the genome size of *B. cookei* is very similar to other members of the Pleosporales, the set of predicted genes in *B. cookei* is notably smaller than many close relatives (Supplementary Fig. S9; Supplementary Table S6). Particularly in the context of other *Bipolaris* species, *B. cookei* had contracted sets of CAZymes, secreted proteins, and candidate effectors. Conversely, *B. cookei* had a substantially larger mitochondrial genome than most other species of Dothideomycetes for which a complete mitochondrial genome is available. The proliferation of introns and ORFs encoding putative homing endonucleases were primarily responsible for the expanded size. Fungal mitochondria usually contain a set of 14 conserved protein-encoding genes required for electron transfer and oxidative phosphorylation^24,49^. However, exceptions have been previously noted. More precisely, the absence of the ATP synthase genes *atp8* and *atp9* was also observed in the *B. cookei* close relatives *Stagonospora nodorum* ^50^ and *Shiraia bambusicola* ^51^, the only Pleosporales members with mitochondrial genomes published to date. This result supports the hypothesis that the absence of *atp8* and *atp9* is a common genomic feature among members of the Pleosporales. Interestingly, *B. cookei* strain LSLP18.3 does not contain a G143A mutation within the mitochondrial *cytochrome b* (*cob*) gene, which is associated with resistance to QoI fungicides^25,52^. At the time strain LSLP18.3 was isolated (2009), fungicides were not commonly applied to sorghum grown in Arkansas, although applications of foliar fungicides on grain sorghum in Arkansas have become increasingly common in recent years. Resistance to QoI fungicides recently emerged in related Dothidiomycetes pathogens of soybean (e.g., *Cercospora sojina* and *Cercospora c. f. flagellaris*) and spread rapidly throughout the southeastern U.S.^53,54^. The finished mitochondrial genome of *B. cookei* will help facilitate future population-level studies to explore the potential emergence of fungicide resistance in this pathosystem.

Pathogens must adapt rapidly to changing environments in order to win ongoing evolutionary arms races with their hosts. The genome sequence of *B. cookei* provides evidence for diverse mechanisms promoting genetic variability and genome evolution. Regarding sexual reproduction, *B. cookei* strain LSLP18.3 possesses an ortholog of *MAT-1* that lacks obvious evidence of inactivation associated with RIP mutations, transposon insertion, or other disruptive mutations. The presence of a presumably functional *MAT-1* mating-type locus indicates the potential for heterothallic sexual reproduction in *B. cookei*, which would be consistent with other closely related *Bipolaris* species^55–57^. Sexual recombination is a central driver of adaptation among many plant pathogenic fungi by creating new combinations of genes and alleles involved in pathogenesis^58^. Byproducts of sexual recombination can also modify genome architecture by inducing genome modifications and rearrangements, such as duplications, inversions, and deletions^59,60^. In plant pathogenic fungi, the ability to induce changes in gene content and genomic architecture is a key component of rapid adaptive evolution^60^. Mobile genetic elements such as transposons are associated with genomic rearrangements and the creation of novel genes and alleles in many plant pathogenic fungi, and specific selective advantages have been documented to result from such genomic modifications^61^. Similar to the genomes of many other plant pathogenic fungi, the genome of *B. cookei* contains a large number of taxonomically diverse transposable elements. The substantial enrichment of SM backbone genes and SSPs near repetitive elements, compared to a negative association of housekeeping genes and other predicted elements of the core genome, is consistent with the presence of ‘repetitive islands’, in which pathogenicity-associated genes are enriched and isolated from components of the core genome^61^. The dynamic nature of such repetitive islands is associated, in part, through genome defense mechanisms such as RIP mutations^62^. Although RIP presumably evolved as a mechanism for fungi to inactivate duplicated sequences in their genomes, the sequences of single-copy genes in close proximity to repetitive elements can be altered via RIP slippage^61,63^, thus leading to the generation of novel alleles. Considering the widespread distribution of repetitive elements in the *B. cookei* genome, and their notably proximity to secondary metabolite genes and candidate effectors, repetitive elements are a plausible source of genomic variability and evolution in *B. cookei*. Yet another mechanism of fungal genome diversification is horizontal gene transfer (HGT), in which genetic material is exchanged between reproductively isolated organisms^64^. Circumstantial evidence for horizontal gene transfer in *B. cookei* is provided by the NR-PKS gene Bc_10041 and the hypothetical gene Bc_10331. Homologs of Bc_10041 and Bc_10331 were present in *Colletotrichum sublineolum*, yet absent from other sequenced *Bipolaris* species, which is inconsistent with a pattern of vertical inheritance from a common ancestor. Because *B. cookei* and *C. sublineolum* are both pathogens of the same host, share overlapping geographical distributions, and have been noted to co-infect individual sorghum leaves, populations of both pathogens have presumably been in more than adequate physical proximity for HGT events to have occurred.

At the current time, whether *B. cookei* utilizes a hemibiotrophic or necrotrophic infection strategy is not fully resolved. Most plant pathogenic members of *Bipolaris* are generally considered to be necrotrophs^15,35^, although a few hemibiotrophic species are postulated to exist within the genus^16^. When considered in the context of the limited histopathological analyses of *B. cookei* infection^12^, the genome sequencing and analysis presented in this study lends support to the hypothesis that *B. cookei* is a necrotrophic pathogen. In particular, available information about gene content, such as the reduced set of candidate effectors, and gene expression are mostly consistent with a necrotrophic growth habit. The *in planta* expression set examined in this study was derived from an early stage of the infection process (24 hr after inoculation), at which time hemibiotrophs are typically initiating latent (asymptomatic) infections. As such, hemibiotrophic pathogens are thought to rely more heavily on suites of effectors that manipulate host defense reactions at early stages of infection, rather than phytotoxic secondary metabolites. However, genes for secondary metabolite production or detoxification in some hemibiotrophic fungi have been previously reported upregulated at early stages of infection^65,66^. Furthermore, it is important to note that the distinction between a necrotroph and a hemibiotroph is currently predicated on the existence of a consistent, definable latent phase before the visible onset of necrosis^35,67^, rather than a specific set of genetic or biochemical criteria. Thus, if the period between inoculation and symptom expression is compressed, as has been reported for *B. cookei* ^12^ the potential existence of an unusually short-lived hemibiotrophic phase cannot fully be discounted. Future histopathological experiments utilizing cell biology approaches, in conjunction with extensive transcriptional profiling, will be required to more conclusively define the infection strategy utilized by *B. cookei*.

The known host range of *B. cookei* is limited to certain members of the grass family (Poaceae), specifically species within the genera *Sorghum* and *Zea* ^9^. The current study provides a degree of insight into potential mechanisms underlying host specificity. In other fungal pathosystems, host specific toxins (HSTs) are important components of host-specific necrotrophy^68,69^. The *B. cookei* LSLP18.3 draft genome contains a substantial number of secondary metabolism genes and clusters, some of which have not yet been described in other fungal genome sequencing projects and thus are potentially unique to *B. cookei*. Interestingly, the SM backbone gene Bc_10041 is taxonomically restricted to *B. cookei* among *Bipolaris* genomes sequenced to date, yet an ortholog is present in the genome of the sorghum pathogen *C. sublineolum*. An intriguing possibility is that one or more metabolites requiring Bc_10041 for their biosynthesis could conceivably function as HSTs on sorghum. The gene expression profiling data analyzed in this study also suggest the potential existence of HSTs in this pathosystem. Among the 37 genes highly expressed *in planta* as opposed to *in vitro*, the majority encoded genes implicated in secondary metabolism, as well as some that appear to be involved in modification or detoxification of host phytoalexins. At this time, it is not clear whether *B. cookei* might utilize a suite of SM-derived HSTs, or if it may instead utilize a limited number of HSTs in conjunction with an arsenal of broad-spectrum necrosis-inducing toxins. Additionally, given that HSTs can be proteins rather than SMs, such as ToxA^70^, the potential contribution of diverse HSTs throughout various stages of the infection process is worthy of further investigation. When considering the potential roles of HSTs in host-specific necrotrophy, much less clear is the potential role of effectors, e.g., pathogen-derived proteins that modulate host defense. It is conceivable that necrotrophs could utilize effectors to manipulate host defense responses in such a way to accelerate cell death, which would be advantageous for pathogen growth. Gene Bc_06468 is particularly interesting as a candidate gene involved in necrotrophic pathogenesis. Although it may have a relatively conventional function such as suppressing host defense responses, it could potentially have a biochemical function related to the induction or maintenance of necrotrophy. However, its function is difficult to infer based on its structure, which contains no conserved domains, or its taxonomic distribution; putative orthologs were found only in related *Bipolaris* spp., *S. turcica*, *P. tritici-repentis*, and species of the *F. oxysporum* complex. Thus, functional characterization of Bc_06468 and other genes underlying pathogenesis will be required to clarify the molecular basis of host-specific necrotrophy in *B. cookei*.

In summary, this work presented a comprehensive analysis of genomic features in *B. cookei* and expression analysis of *B. cookei* genes induced at early stages of infection. The results provided important and novel insights into the molecular basis of target leaf spot on sorghum, and highlighted several candidate pathogenicity-related genes that were taxonomically restricted among *Bipolaris* species. Lastly, the draft genome assembly of *B. cookei* will serve as a valuable genomic resource for functional genomics studies and population genetics in this organism.

## Materials and Methods

### Fungal strain and culture conditions

Wild-type strain LSLP18 of *B. cookei* was isolated from a diseased sorghum plant collected at the University of Arkansas Lon Mann Cotton Research Station in Lee County, Arkansas, during the 2009 growing season. The isolate was selected for genome sequencing due to a high level of virulence and stable growth in routine laboratory culture. To confirm Koch’s postulates, the sorghum line BTx623 was grown in a growth chamber maintained at 25 °C under a 12 h photoperiod. A conidial suspension (5 × 10^4^ conidia ml^−1^) in 0.01% Tween-20 was atomized onto the adaxial surface of sorghum leaves three weeks after planting until just before runoff. Inoculated plants were placed in transparent plastic bags and incubated in a growth chamber at 25 °C and near 100% relative humidity under a 12 h photoperiod. After 48 h, plants were removed from the bags and maintained in a growth chamber at 25 °C under a 12 h photoperiod until symptoms developed. Working cultures were maintained on V8 agar medium^71^. The fungus was stored as colonized agar in 30% (v/v) glycerol at −80 °C. For DNA isolation, a single-spore isolate (LSLP18.3) was derived from LSLP18 and grown in yeast extract peptone dextrose liquid medium^72^ for four days at 25 °C with shaking at 150 RPM.

For RNA sequencing, cultures of *B. cookei* were grown on eight different conditions on solidified media plates overlaid with cellophane (V8 juice agar at constant darkness and constant light; 0.2× strength potato dextrose agar; complete medium agar; minimal medium agar; minimal medium pH 3; minimal medium pH 8; minimal medium with ammonium as nitrogen source), liquid minimal medium, and yeast extract peptone dextrose medium^72^. The tissue from each culture was harvested five days after inoculation, flash frozen immediately, and ground in liquid nitrogen. The ground tissue was either used immediately for RNA extraction or stored at −80 °C.

### DNA and RNA extraction and sequencing

For DNA isolation, strain LSLP18.3 was grown as described above. Tissue was collected by filtration, frozen in liquid nitrogen, and ground in a pre-chilled mortar and pestle containing sterile glass beads (0.5 mm) (Research Products International, Mt. Prospect, IL, USA). Genomic DNA was isolated with a modified cetyltrimethylammonium bromide (CTAB) method^71^. DNA was further purified with a Qiagen Genomic-tip 500/G column (Qiagen, Germantown, MD, USA) following the manufacturer’s recommendations. DNA quality and quantity was determined by agarose gel electrophoresis and a NanoDrop ND-1000 Spectrophotometer (Thermo Fisher Scientific, Waltham, MA, USA). Genome sequencing was performed with a hybrid sequencing approach. Library preparation (fragment size = 500 bp) and sequencing (one lane, paired-end, 100 bp read length) was performed by BGI Americas (Cambridge, MA, USA) with an Illumina HiSeq. 2000 Sequencing System (Illumina Inc., San Diego, CA, USA). Additionally, library preparation (library size = 3–10 kb) and sequencing (two SMRT cells) was performed by the Yale Center for Genome Analysis (Orange, CT, USA) with a PacBio RS II Sequencing System (Pacific Biosciences, Menlo Park, CA, USA).

For RNA isolation, strain LSLP18.3 was grown as described above. Tissue was collected, frozen in liquid nitrogen, and ground in a pre-chilled mortar and pestle containing sterile glass beads (0.5 mm) (Research Products International). Total RNA was isolated with a Direct-zol RNA MiniPrep Kit (Zymo Research, Irvine, CA, USA) following the manufacturer’s recommendations. DNA quality and quantity was determined by agarose gel electrophoresis and a NanoDrop ND-1000 Spectrophotometer (Thermo Fisher Scientific). Total RNA from each condition was mixed in equal amounts. Pooled total RNA (10 μg) was subjected to mRNA enrichment with a MagJET mRNA Enrichment Kit (Thermo Fisher Scientific). Ribosomal RNA-free mRNA was fragmented with RNase III (New England Biolabs, Ipswich, MA, USA) at 37 °C for 5 minutes. First strand cDNA was synthesized from the fragmented mRNA with random hexamers (Integrated DNA Technologies, Inc., Coralville, IA, USA) and M-MLV Reverse Transcriptase (Promega, Madison, WI, USA), and second strand synthesis was performed with a NEBNext mRNA Second Strand Synthesis Module (New England Biolabs). End repair was performed on the second strand DNA with a NEBNext End Repair Module (New England Biolabs). Following end repair, sequencing adapters were ligated to the double stranded cDNA with a NEBNext Fast DNA Fragmentation & Library Prep Set for Ion Torrent (New England Biolabs). Size selection for 480 bp was performed with an Agencourt AMPure XP kit (Beckman Coulter, Brea, CA, USA). A final PCR enrichment step was performed following the recommendations provided with the NEBNext Fast DNA Fragmentation & Library Prep Set for Ion Torrent to amplify fragments containing ligated adapters at both ends. The size distribution and quality of the library was determined with an Agilent Tapestation 2200 D1K (Agilent Technologies, Santa Clara, CA, USA). The library was sequenced on an Ion Torrent Personal Genome Machine System with an Ion 318 Chip Kit v2 (Thermo Fisher Scientific).

### Genome assembly and repetitive DNA identification

The draft genome of *B. cookei* was assembled based on a custom hybrid strategy (Supplementary Fig. S1). The Illumina reads were corrected with Bayes Hammer within SPAdes v3.1 assembler^73^. Corrected Illumina reads were used to correct the PacBio reads with LoRDEC v0.2^74^. Illumina and PacBio reads were analyzed with SPAdes v3.1, which produced a raw draft assembly. Contigs from the draft assembly were merged into scaffolds with successive iterations of SSPACE-Standard v3.0^75^ and AHA from smrtanalysis suite 2.3 (http://www.pacb.com/support/software-downloads/). Genomic regions containing gaps were filled with GapFiller v1.11^76^ and PBJelly v14.7.14^77^.

A homology search with genes from the *Stagonospora nodorum* mitochondrial genome^50^ identified one assembled scaffold (scaffold75) corresponding to the *B. cookei* mitochondrial genome. Illumina and PacBio reads were mapped to the *B. cookei* assembly with Bowtie v2.2.9^78^ and BLASR v1.3.1^79^, respectively, and the reads that mapped to scaffold75 were given to SPAdes to produce a new assembly, which was improved with SSPACE-Standard v3.0. This produced a single contig with overlapping ends.

Repetitive DNA sequences in the *B. cookei* genome were identified with RepeatScout v1.0.5^80^ using an *l*-mer value of 13, and filtered with the accessory script *filter-stage-1.prl*. Repetitive elements that appeared less than 10 times in the genome were filtered out with the accessory script *filter-stage-2.prl* and RepeatMasker open-4.0.5 (http://www.repeatmasker.org). Annotation of transposable elements was performed with TransposonPSI (http://transposonpsi.sourceforge.net). The Repeat-Induced Point (RIP) mutation index (TpA/ApT) was calculated with the *dinucleotide Frequency* function within the R package Biostrings v2.44.2^81^ based on a sliding window approach with window size of 300 bp and step size of 50 bp. The distance of genes to repetitive elements was calculated with the function *closest* within BEDtools v2.26^82^.

### Gene prediction and functional annotation

For gene prediction, RNA sequencing reads were first mapped to the draft genome with GSNAP v2014-10-09^83^ with the splicing option (*-N*) enabled. Then, mapped RNA-seq reads were analyzed with Cufflinks v2.2.1^84^ to reconstruct transcripts with default parameters. Gene models were predicted with Maker pipeline v2.32.6^85^ with the reconstructed transcripts from *B. cookei* as EST evidence, and protein sequences from *B. maydis* C5 and *B. zeicola* (http://genome.jgi.doe.gov/programs/fungi/index.jsf) as protein homology evidence. Gene models were selected with the accessory script *Maker2zff* within the Maker software package with default parameters to train the *ab initio* predictors SNAP v2006-07-28^86^ and Augustus v3.0.2^87^. A new set of gene models was predicted with Maker adjusted to use SNAP and Augustus simultaneously with the *B. cookei* reconstructed transcripts, and the protein sequences from *B. maydis* C5 and *B. zeicola*. Gene completeness was assessed with BUSCO software v2^88^ using protein assessment mode (-*m* protein) and the Ascomycota lineage (-*l*).

To functionally characterize genes, predicted protein sequences were queried against the NCBI nr database (update 03/2016) with BLAST v2.2.31^89^ and an e-value < 1e-5. BLAST results were analyzed with Blast2GO v3.0^90^ to attribute functional descriptions and GO terms for each protein. Mitochondrial genes were identified and annotated with MFannot (http://megasun.bch.umontreal.ca/cgi-bin/mfannot/mfannotInterface.pl) and MITOS2^91^. Uncharacterized ORFs in the mitochondrial genome were annotated with Blast2GO as described previously.

The mating type of *B. cookei* was determined with BLAST homology searches using as queries the mating genes *MAT-1* (GenBank accession: CAA48464) and *MAT-2* (GenBank accession: CAA48465) from *B. maydis* ^92^.

Carbohydrate-active enzymes were identified with a local installation of dbCAN v5.0^93^ and HMM v3.1b1 (http://hmmer.org). Polyketide synthases (PKSs), nonribosomal peptide synthatases (NRPSs), and dimethylallyl transferases (DMTs) were identified with SMURF^39^, and terpene synthases (TSs) were identified with antiSMASH v3.0.5^94^. PKSs were queried against the NCBI conserved domain database (https://www.ncbi.nlm.nih.gov/Structure/cdd/wrpsb.cgi) for further categorization. PKSs containing a starter unit:ACP transacylase (SAT) domain were classified as non-reducing PKSs, those containing an enoyl reductase (ER) domain were classified as highly reducing PKSs, and those that lacked both SAT and ER domains were classified as partially-reducing PKSs.

To identify putative secreted proteins, all predicted proteins were evaluated for the presence of signal peptides with SignalP v4.1^95^ and TargetP v1.1^96^. Proteins containing a signal peptide as well as two or more transmembrane domains, as determined with TMHMM v2.0^97^, or a glycosylphosphatidylinositol (GPI) anchor, as determined with PredGPI^98^, were categorized as plasma membrane-associated rather than secreted. The remaining proteins (comprising the putative secretome) were further categorized into secreted proteases, lipases and peroxidases by performing BLAST searches against the MEROPS database v11^99^, Fungal Peroxidase Database^100^, and Lipase Engineering Database^101^, respectively, with a maximum e-value of 1e-5. Secreted proteins classified as effectors according to EffectorP v1.0^102^, or containing less than 300 amino acids with at least 2% of predicted residues corresponding to cysteine, calculated with EMBOSS package v6.6^103^, were considered to be candidate effectors.

### Gene expression and comparative genomic analyses

RNA-seq reads from sorghum leaves infected with *B. cookei* 12 h and 24 h post-inoculation^21^ were combined and mapped to the *B. cookei* genome assembly with GSNAP v2014-10-09, and then compared with RNA-seq data obtained from *B. cookei* grown on various defined culture media *in vitro*. Reads mapped to each predicted ORF were counted with the function *coverage* within BEDtools v2.26. Genes with more than three *in planta* RNA-seq reads and zero *in vitro* RNA-seq reads were considered exclusively expressed *in planta*. In addition, genes with a ratio of at least 4:1 regarding *in planta* and *in vitro* RNA-seq reads, respectively, were also considered induced during sorghum infection.

Comparative genomic analyses with other *Bipolaris* species were performed with the genomes of *B. maydis* (=*Cochliobolus heterostrophus*) C5, *B. sorokiniana* (= *C. sativus*) ND90Pr, *B. zeicola* (=*C. carbonum*) 26-R-13, *B. oryzae* (= *C. miyabeanus*) ATCC 44560, and *B. victoriae* (= *C. victoriae*) FI3^15,35^, obtained from the JGI website (http://genome.jgi.doe.gov/programs/fungi/index.jsf).

### Data availability

This Whole Genome Shotgun project has been deposited at DDBJ/ENA/GenBank under the accession NRSV00000000. The version described in this paper is version NRSV01000000. The complete mitochondrial genome has been deposited at GenBank under accession MF784482. RNA-seq data obtained from culture media conditions have been deposited at NCBI sequence read archive (SRA) under accession SRR5957114.

## Electronic supplementary material

Supplementary figures

Supplementary tables
