# Supplementary material for: The genome sequence of Bipolaris cookei reveals mechanisms of pathogenesis underlying target leaf spot of sorghum
Source: Sci Rep. 2017 Dec 8;7:17217. doi: 10.1038/s41598-017-17476-x (PMC5722872; doi:10.1038/s41598-017-17476-x)
Supplement: Supplementary file 1 — Supplementary figures [file 41598_2017_17476_MOESM1_ESM.pdf]

Supplementary figures

Title: The genome sequence of *Bipolaris cookei* reveals mechanisms of pathogenesis underlying target leaf spot of sorghum.

Authors: Alex Z. Zaccaron and Burton H. Bluhm*

Affiliation: Department of Plant Pathology, University of Arkansas, Division of Agriculture, Fayetteville, AR 72701, USA.

*For correspondence: E-mail bbluhm@uark.edu; Tel. +1-479 575 2677; Fax +1 479 575 7601

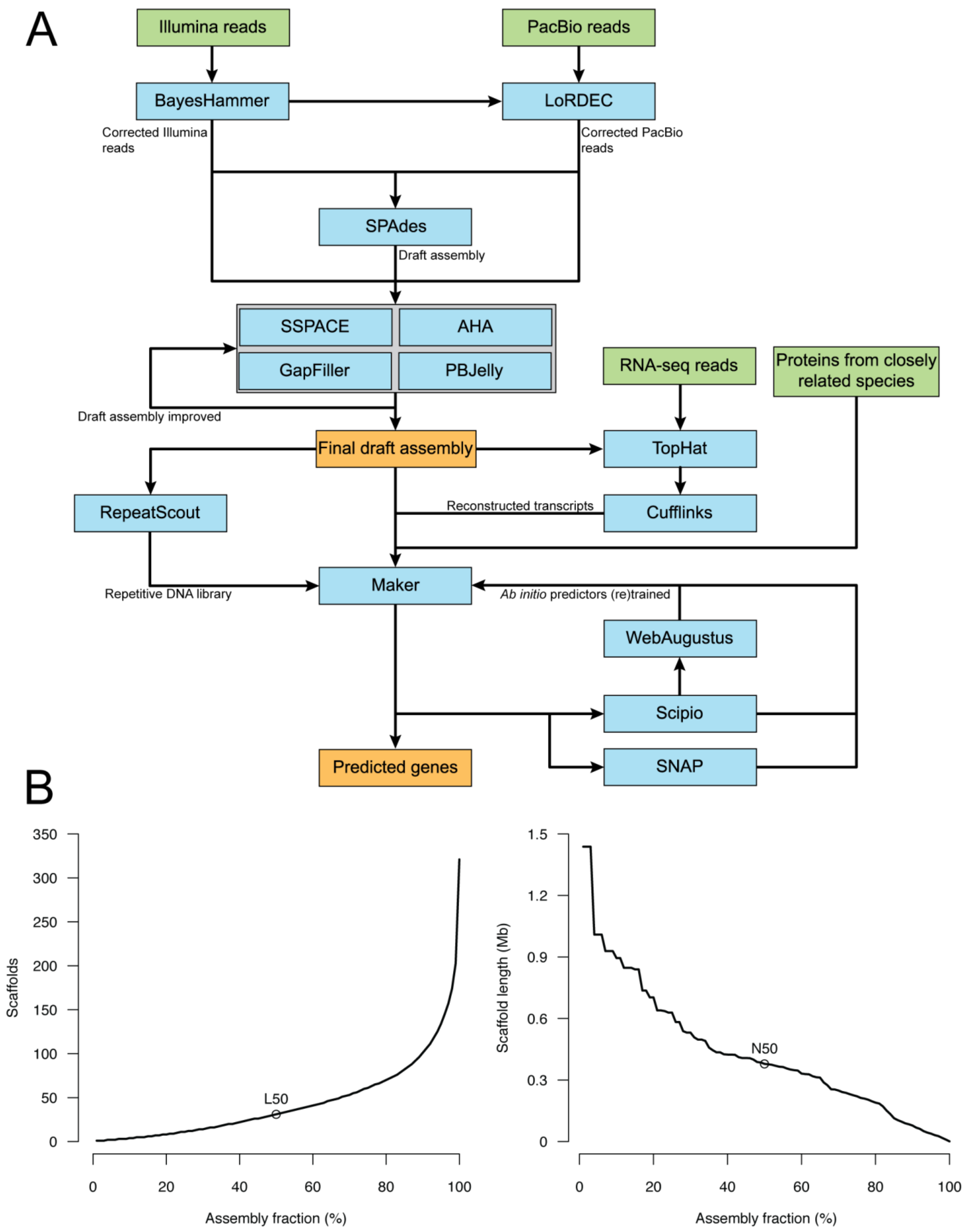
A
Illumina reads
PacBio reads
BayesHammer
LoRDEC
Corrected Illumina reads
Corrected PacBio reads
SPAdes
Draft assembly
SSPACE
AHA
GapFiller
PBJelly
Draft assembly improved
Final draft assembly
RNA-seq reads
Proteins from closely related species
TopHat
RepeatScout
Reconstructed transcripts
Cufflinks
Maker
Repetitive DNA library
Ab initio predictors (re)trained
WebAugustus
Scipio
Predicted genes
SNAP
B
Scaffolds
L50
Assembly fraction (%)
Scaffold length (Mb)
N50
Assembly fraction (%)

Supplementary Fig 1: *B. cookei* genome assembly pipeline and overview. (A) Pipeline used to assembly and predict the genes of *B. cookei*. Data given as input is represented as green rectangles, computational tools as blue rectangles, and major output of the pipeline as orange rectangles. (B) Variation of the L and N values for different fractions of the assembly. The L value is the minimum number of scaffolds to represent a fraction of the genome assembly, and the N value is the length of the shortest of such scaffolds.

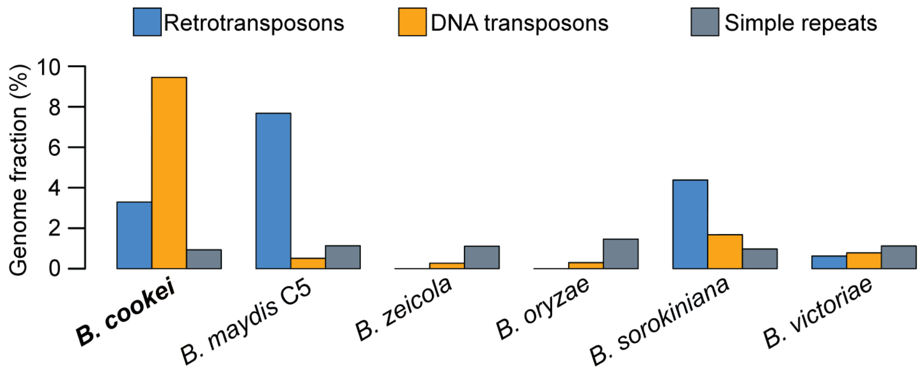

Supplementary Fig. 2: Retrotransposon, DNA transposon, and simple repeat content in *B. cookei* and other *Bipolaris* ssp. The bar chart shows the approximate fraction of the whole genome covered by retrotransposons and DNA transposons, according to TransposonPSI annotation (http://transposonpsi.sourceforge.net), and simple repeats, according to RepeatMasker (http://www.repeatmasker.org).

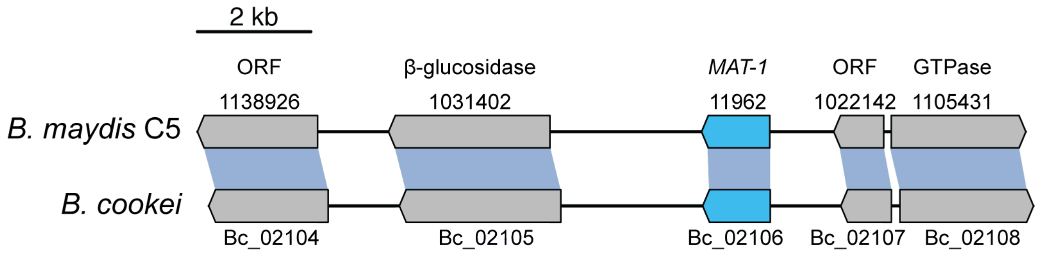

Supplementary Fig. 3: *MAT-1* locus in *B. maydis* C5 and *B. cookei*. JGI protein IDs and their respective functional description are shown for *B. maydis* C5 genes. At the amino acid level, the ORF 1138926 and Bc_02104 are 84% identical, the beta-glucosidase 1031402 and Bc_02105 are 97% identical, the *MAT-1* is 87% identical, the ORF 1022142 and Bc_02107 are 95% identical, and the GTPase 1105431 and Bc_02108 are 93% identical.

```
                          1                                                   50
B. cookei LSLP18          MRLFKSHPIL RLANSYLVDS PQPMNLSYMW NFGSLLAFCL VMQIVTGVTL
C. Beticola 98-M-10       ~~~~~~~~~~ ~~~~~~~~~~ ~~~~~~~~~~ ~~~~~~~~~~ ~~~~~~~~~~
C. Beticola 11-1027-1     ~~~~~~~~~~ ~~~~~~~~~~ ~~~~~~~~~~ ~~~~~~~~~~ ~~~~~~~~~~

                          51                                                 100
B. cookei LSLP18          AMHYNPSVAE AFNSVEHSIM RDVNNGWLMR YLHSNTASAF FFLVYTHMGR
C. Beticola 98-M-10       ~~~~~~~~~~ ~~~~~~~~~~ ~~~~~~~~~~ ~~~~~~ASAF FFLVYLHVGR
C. Beticola 11-1027-1     ~~~~~~~~~~ ~~~~~~~~~~ ~~~~~~~~~~ ~~~~~~ASAF FFLVYLHVGR

                          101                                                150
B. cookei LSLP18          NMYYGSYRAP RTLVFTIGVV IFILMMAKEA SLYVLPYGQM STWGEFCITN
C. Beticola 98-M-10       GLYYGSYKAP RTLVWTIGTI ILVLMMATAF LGYVLPYGQM SLWGATVITN
C. Beticola 11-1027-1     GLYYGSYKAP RTLVWTIGTI ILVLMMATAF LGYVLPYGQM SLWAATVITN

                          151                                                200
B. cookei LSLP18          TMSAIPWVGQ DIVESYQHKC SVNNATLNRF FSLHFVLPFV LAALATMHLI
C. Beticola 98-M-10       LMSAIPWVGQ DIVEFLWGGF SVNNATLNRF FALHFVLPFV LAALALMHLI
C. Beticola 11-1027-1     LMSAIPWVGQ DIVEFLWGGF SVNNATLNRF FALHFVLPFV LAALALMHLI

                          201                                                250
B. cookei LSLP18          VLHDTAGSGN PLGVSGNYDR MPFAPYLMFK DTITIFAFMF VLSLFVFFMP
C. Beticola 98-M-10       ALHDSAGSGN PLGVSGNYDR LPFAPYFIFK DLITIFLFII VLSVFVFFMP
C. Beticola 11-1027-1     ALHDSAGSGN PLGVSGNYDR LPFAPYFIFK DLITIFLFII VLSVFVFFMP

                          251                                                300
B. cookei LSLP18          NVLGDSENYV VANPMQTPAA IVPKHDTTPF YAMLRSIPNK LLGVMAMFAA
C. Beticola 98-M-10       NVLGDSENYV VANPMQTPPA IVPEWYLLPF YAILRSIPNK LLGVIAMFSA
C. Beticola 11-1027-1     NVLGDSENYV VANPMQTPPA IVPEWYLLPF YAILRSIPNK LLGVIAMFSA
```

```
                         301                                                350
B. cookei LSLP18         ITILTTLPVT DVSRSRGMQF RPLSKAAFFA FVANFLILMQ LGAKHVESPF
C. Beticola 98-M-10      ~~~~~~~~~~ ~~~~~~~~~~ ~~~~~~~~~~ ~~~~~~~~~~ ~~~~~~~~~~
C. Beticola 11-1027-1    ~~~~~~~~~~ ~~~~~~~~~~ ~~~~~~~~~~ ~~~~~~~~~~ ~~~~~~~~~~

                         351                                   386
B. cookei LSLP18         IEFGQMSTVL YFSYFTFVMY GVTVTENTFV DLRHKK
C. Beticola 98-M-10      ~~~~~~~~~~ ~~~~~~~~~~ ~~~~~~~~~~ ~~~~~~
C. Beticola 11-1027-1    ~~~~~~~~~~ ~~~~~~~~~~ ~~~~~~~~~~ ~~~~~~
```

Supplementary Fig. 4: Protein sequence alignment of *cytochrome b* (*cob*) from *B. cookei* and *Cercospora beticola*. The point mutation G143A associated with resistance to QoL fungicides is highlighted. *C. beticola* isolate 98-M-10 (GenBank accession JQ619932) does not have the mutation G143A, while *C. beticola* isolate 11-1027-1 (GenBank accession JQ619933) has the mutation G143A.

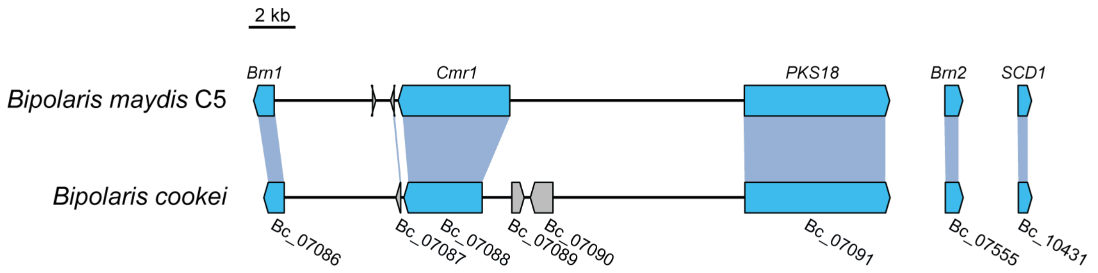

Supplementary Fig. 5: Genes involved in melanin biosynthesis in *B. maydis* C5 and *B. cookei*. Genes predicted to be involved in melanin biosynthesis are drawn in blue. *Brn1* encodes a 1,3,8-trihydroxynaphthalene (T3HN) reductase, *Cmr1* encodes a transcription factor, *PKS18* encodes a polyketide synthase, *Brn2* encodes a 1,3,6,8-tetrahydroxynaphthalene (T4HN) reductase, and *SCD1* encodes a scytalone dehydratase. The genes *Brn2* and *SCD1* are in different locations in the genome of both species. Genes in grey are hypothetical. JGI protein ID of the *B.*

maydis C5 genes, from left to right: *Brn1*: 1018608; hypothetical: 1018609; hypothetical: 1018611; *Cmr1*: 1208764; *PKS18*: 30478; *Brn2*: 1022295; and *SCD1*: 1024460.

```
Ecp6           1 MQSMILFAAALMGAAVNGFVLPRTDDPDCET--KATDCGSTSNIKYTVVK     48
                   |:|.:....|::.|:|:.    |....||::  ....|...|...||:|.
Bc_04981       1 MKSTLFAIVAVLAASVSA----RPTKTDCKSPYPNVSCKPLSLKNYTIVS     46

Ecp6          49 GDTLTSIAKKFKSGICNIVSVNKLANPNLIELGATLIIPENC-SNPDNKS     97
                   |||||:||..|.||.||||::|.::||:||..|..:.:|.|| :..|..|
Bc_04981      47 GDTLTTIADHFGSGACNIVALNNISNPDLIFPGELVTVPANCTATVDKTS     96

Ecp6          98 CVSTPAEPTET--CVPGL---PGSYTIVSGDTLTNISQDFNITLDSLIAA    142
                   |:|...:.|.|  ||.||   |..|.::..||.|.|:.:|::.||:|..|
Bc_04981      97 CLSNAPQATGTQDCVKGLSVNPPVYQVIPKDTFTLIANNFDLKLDALENA    146

Ecp6         143 NT-QIENPDAIDVGQIITVPVCPSSQCEAVGTYNIVAGDLFVDLAATYHT    191
                   |. :..:.|||..|....:|||....|... .|.||:||.|..:|.....
Bc_04981     147 NKGRFASFDAIFAGNTTIIPVCQGCSCYDT-KYTIVSGDTFGAIAKNSSI    195

Ecp6         192 TIGQIKALNNNVNPSKLKVGQQIILPQDCKNVTTAVA    228
                   |||||:|.|....|.:|::||.|..|     |.:.||
Bc_04981     196 TIGQIEAANPGQIPEQLQIGQVINRP-----VCSCVA    227
```

Supplementary Fig. 6: Protein sequence alignment of *Ecp6* from *Cladosporium fulvum* and Bc_04981 from *B. cookei*. Highlighted regions correspond to LysM motifs, according to dbCAN v5.0 (http://csbl.bmb.uga.edu/dbCAN/). UniProt accession number of *Ecp6*: B3VBK9.

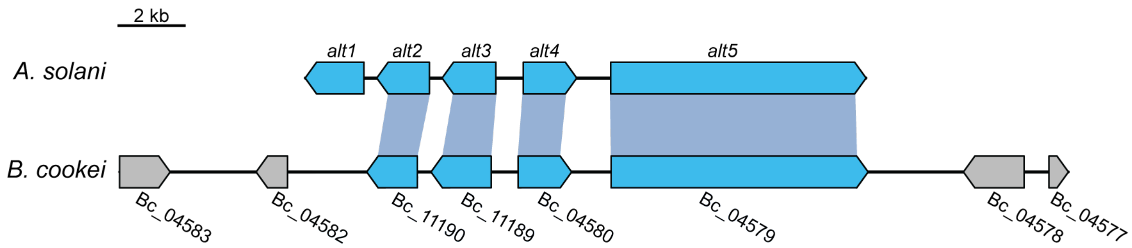

Supplementary Fig. 7: Alternapyrone gene cluster from *Alternaria solani* and the corresponding homolog cluster in *Bipolaris cookei*. The genes *alt1*, *alt2*, and *alt3* encode cytochrome P450s, *alt4* is a FAD-dependent oxygenase/oxidase, and *alt5* is a polyketide

synthase. Amino acid identity of *alt2* and Bc_11190: 82%; *alt3* and Bc_11189: 87%; *alt4* and Bc_04580: 76%; *alt5* and Bc_04579: 82%. GenBank accession of *alt1-5*: AB120221.1.

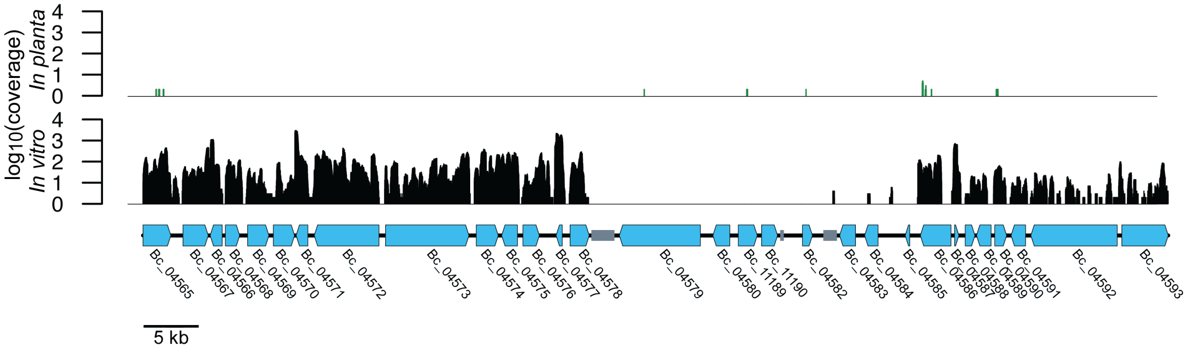

Supplementary Fig. 8: Region of the *Bipolaris cookei* genome where the homolog cluster for alternapyrone biosynthesis is located. Genes are represented as blue polygons, and repetitive DNA as grey rectangles. *B. cookei* homologs of the genes for alternapyrone biosynthesis: Bc_04579, Bc_04580, Bc_04589, and Bc_11190. Coverage of RNA-seq data from different culture media conditions (*in vitro*) and from sorghum leaves infected with *B. cookei* (*in planta*) is shown in $\log_{10}$ scale.

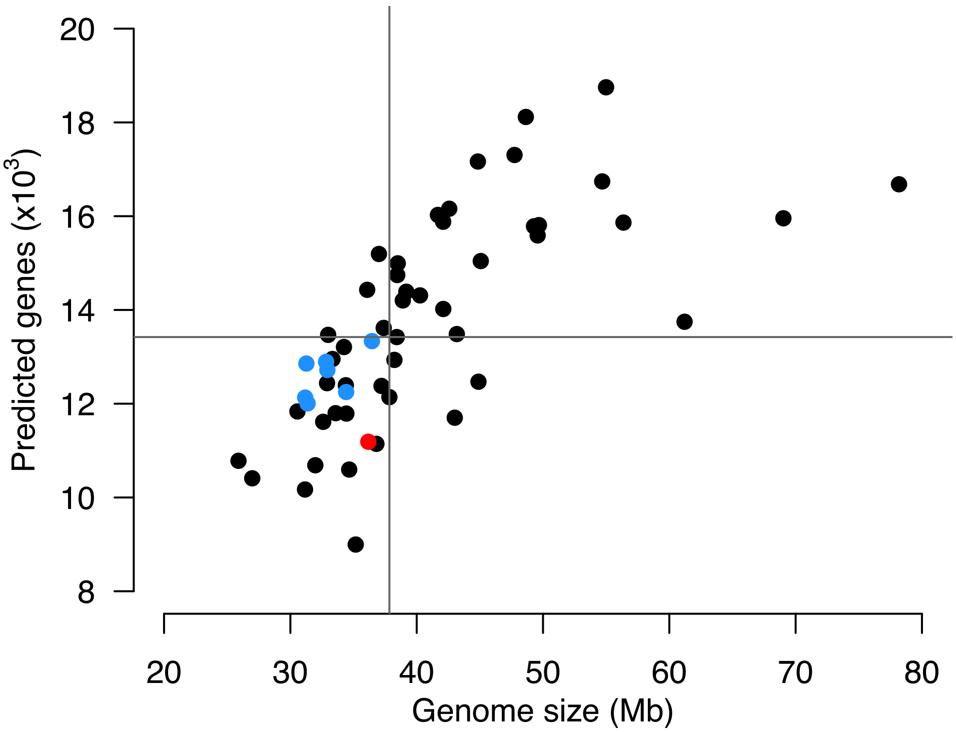

Supplementary Fig. 9: Chart showing the genome size and number of predicted genes from *B. cookei* and other 55 members of the Pleosporales. *B. cookei* is shown as a red point. Other *Bipolaris*/*Curvularia* ssp. are shown as blue points. Median values are shown as grey lines. Values were obtained from JGI website (http://genome.jgi.doe.gov/pleosporales/pleosporales.info.html). Numbers can be seen in Table S6.
